# Environmentally friendly comprehensive hydrometallurgical method development for neodymium recovery from mixed rare earth aqueous solutions using organo-phosphorus derivatives

**DOI:** 10.1038/s41598-020-74041-9

**Published:** 2020-10-09

**Authors:** Verónica Cristina Arellano Ruiz, Rambabu Kuchi, Pankaj Kumar Parhi, Jin-Young Lee, Rajesh Kumar Jyothi

**Affiliations:** 1 Convergence Research Center for Development of Mineral Resources (DMR), Korea Institute of Geoscience & Mineral Resources (KIGAM), Daejeon, 34132 Korea; 2 Department of Resources Recycling, University of Science and Technology (UST), Daejeon, 34113 Korea; 3 Department of Chemistry, Fakir Mohan University, Balasore, Odisha 756 089 India

**Keywords:** Environmental sciences, Environmental chemistry, Environmental impact

## Abstract

Rare earth elements (REEs) have obtained a greatest significant in human lives owing to their important roles in various high technology applications. The present method development was deal technology important REEs such as neodymium, terbium and dysprosium, selective extraction with possible separation and recovery studies, successfully. The chloride mediated mixed aqueous solution containing 1500 mg/L each of REEs such as Nd, Tb and Dy was subjected at selective separation of Nd from other associated REEs. Three organo-phosphorous based commercial extracting agents such as Cyanex 272, PC 88A and D2EHPA, were employed for the extraction, possible separation and recovery of rare earth elements. A comparative extraction behavior of all these three extractants as function of time, pH influence, extractant concentration, temperature and diluents were systematically investigated. The extraction tendency of organo-phosphorus reagents towards the extraction of either of the REEs follows of the sequence as: D2EHPA > PC 88A > Cyanex 272. The thermodynamic behavior of either of the extractants on liquid–liquid extraction processing of REEs was investigated and thermodynamic calculations were calculated and presented. Substantial recovery of neodymium oxalate followed by its calcined product as neodymium oxide was ascertained from XRD study and SEM–EDS analysis.

## Introduction

Dysprosium (Dy), neodymium (Nd), and terbium (Tb) known as part of the rare earth elements (REEs) are vital components in permanent magnets, catalysts, astronomical instruments, coloring glass and ceramics, glass laser, and hybrid engines. Amongst, REEs, Nd is extensively used exclusively in the manufacturing of several high-tech devices such as computers, printers and motors leading to exploitation of it from numerous sources. According to the European commission (year 2017) report^1^, neodymium is one of the most critical REEs with the supply risk in the extraction stage. Furthermore, it is anticipated a high demand for neodymium over the next 25 years^1,2^. Due to the limited deposit of these REEs bearing sources, secondary wastes are becoming potential sources in the present age. Nevertheless, due to the growing demand of neodymium, there has been the unprecedented effort put forward by researchers for its extraction study and with time, there has been a number of processes developed by researchers for recovering neodymium as well as other REEs from these waste (or) the scrap sources^3–15^. The hydrometallurgical processing technology methodologies routed through leaching, solvent extraction (also called liquid–liquid extraction) and precipitation are often preferred as promising techniques for recovering REEs from these secondary wastes including batteries, spent magnets, e-wastes and others which have a number of advantages over pyro-metallurgical route. The prominent features of these techniques are low production costs, the small amount of waste generation, low levels of liberation of noxious gases (SOx, NOx, CO_2_ and CO) ascertained on preventing environmental contamination vis-a-vis a clean separation of targeted REEs. Generally, in hydrometallurgical investigation, first the above secondary phases are subjected to chemical leaching followed by a purification study with either of the methodologies accomplishing liquid–liquid extraction, adsorption/ion exchange for separation and/or precipitation. In the downstream separation processes, the majority of rare earth extractions are followed through solvent extraction technology owing to its widespread and proven attributes. These major processes follow leaching subsequently by solvent extraction and precipitation^16–19^. It has been noticed that during the process development the key challenge is encountered at the downstream stage for separation of the Nd in presence of Dy, Pr and Tb since they possess similar characteristics separation behavior^16–19^.

The solvent extraction (SX) is one of the most significant approach in rare earth separation field over other adopted methods; that is attributed to the usages of numerous organic reagent(s) for effective extraction of target REEs^20^. Nonetheless, the SX process further enables on separation of target REEs up to greater extent in presence of other REEs and/or base metal impurities^15,21^. Therefore, the selection of the appropriate solvent reagent is one of the important factors to ascertain separation^2^. As of now, several commercial reagents including D2EHPA, PC 88A, TOPO, Cyanex 272, TBP, Cyanex 923, Cyanex 921, Cyanex 302 and limited bi-functional ionic liquids are employed for extraction of rare earth elements such as neodymium, terbium and dysprosium from processed aqueous media and /or synthetic solutions.

Mohammadi et al. used D2EHPA and EHEHPA for separation of Dy, Nd and Y where Nd was separated better with D2EHPA than EHEPA^3^. The permanent magnet leach liquor bearing Nd and Dy was systematically investigated on comparative extraction behavior of D2EHPA and PC 88A. Yoon et al. excellent separation (separation factor ~ 247.2) was obtained while using 0.1 mol/L D2EHPA to separate Dy and Nd at extraction stage and during stripping with HCl, maximum stripping yield of 55% Nd and 85% Dy was obtained^4^. Parhi et al. has compared the extraction behavior of D2EHPA and Cyanex 272 towards separation of Nd, Pr from chloride mediated leached solution. Due to the high concentration in the initial Nd solution, saponified D2EHPA was used for effective extraction and enrichment of Nd as well as Pr from permanent magnet leached solution. The chloride based Nd-Fe-B magnet leached solution was subjected for selective separation of Dy from Nd with the separation factor ~ 53.65 at acidic solution pH 1.26^5^. In another study, Padhan et al. has examined the comparison of Cyanex 302, PC 88A and Cyanex 272 from the synthetic solution having 1.0 g/L Nd solution^6^. Kim et al. studied the separation of Nd, Dy, Pr in presence of base metal impurities using ionic liquids (ILs) namely TOGDA and oxide reagent like Cyanex 921, resulting on selective separation of all these REEs by TOGDA^7^. It is noticeable on extraction either of the above REEs using organo-phosphorous reagents in an effective way. In most case studies, separation is performed from the solution bearing binary or unitary REEs systems with limited reports of tertiary systems^8–11^. Moreover, it has also been observed on attainment of low separation factors with most of the adopted solvent reagents. In some studies, more than one similar group reagents and their synergistic approach are also adopted to improve separation behavior of REEs with either of the solvents^12–15^.

Therefore, this present investigation aims to develop a selective recovery and a separation process through solvent extraction. An extensive experimental investigation for comparative behaviors of PC 88A, D2EHPA and Cyanex 272 for separation of Nd from Dy and Tb was systematically investigated. The complexation behavior of organo-phosphorous reagents was ascertained from FT-IR analysis study. The cation exchange mechanism was proposed based on the slope analysis method. A selective separation of Nd was attained at mild pH range with an elevated concentration range of D2EHPA over the other two reagents which has been keenly illustrated. The extraction isotherm and stripping isotherm lead to improve the enrichment of Nd up to 11.7 folds. A green reagent like oxalic acid is employed for effective stripping precipitation of Nd to recover pure neodymium oxalate. The final high pure product of neodymium oxide was characterized and ensured from XRD analysis.

## Result and discussions

### Effect of time on rare earths extraction

The influence of the time in the range of 5 to 90 min extraction of trivalent REEs neodymium, terbium and dysprosium by all the three organo-phosphorus derivatives (D2EPHA, PC 88A, and Cyanex 272) for determining the optimum time required to attain the optimum equilibrium conditions was examined. The experiment was carried out using 1500 mg/L each of REEs mixed solution (Nd, Tb and Dy) from chloride mediated aqueous solution; 0.8 mol/L of either of the extraction agents (D2EPHA, Cyanex 272 and PC 88A). The experiment was carried out at initial pH of aqueous feed solution of 4.0, while temperature of 298 K, phase ratios A/O = 1:1 were kept constant. The obtained experimental results are presented in (Supplementary Data file Fig. S1). It was detected that around ~ 99% extraction of rare earths such as Nd, Tb, and Dy with 0.8 mol/L of D2EPHA, whereas with 0.8 mol/L of PC88A 98% of Dysprosium, 91% of Terbium and 64% of Neodymium were extracted. On the other hand, 76%, 61%, and 27% of Dy, Tb and Nd respectively using 0.8 mol/L of Cyanex 272 were extracted from the synthetic leach liquor in 60 min of contact time. Amongst the extractants, D2EHPA showed faster extraction rate and the extraction equilibrium was reached just after 10 min. It was seen that the extraction trend showed higher extraction rate with D2EHPA followed by PC 88A and then Cyanex 272 at the studied contact time ranges. Contact time and there after the extraction trend remains unaltered. However, to have consistency of achieving efficient extraction for either of the extractants, entire subsequent experimental investigations were chosen to carry out in 60 min of reaction time.

### Effect of equilibrium pH

The pH of the solution plays a critical role on the speciation of REEs in solvent extraction (liquid–liquid extraction) process. In this investigation, studied metals such as Dy, Tb and Nd do exist in trivalent form at acidic pH range of the solution, though extraction behaviour may vary from metal to metal with different separation factors (SFs). Therefore, to investigate in detailed, the pH variation study of the solution has been systematically investigated for extraction of Dy, Tb and Nd using all three P based extractants. The pH of mixed REEs synthetic leach liquor was changed from 1.0 to 5.0 and where the other parameters such as 0.8 mol/L of extractant concentration, equilibrium time 60 min and phase ratio (A/O) is 1 were kept constant.

**Figure 1:**
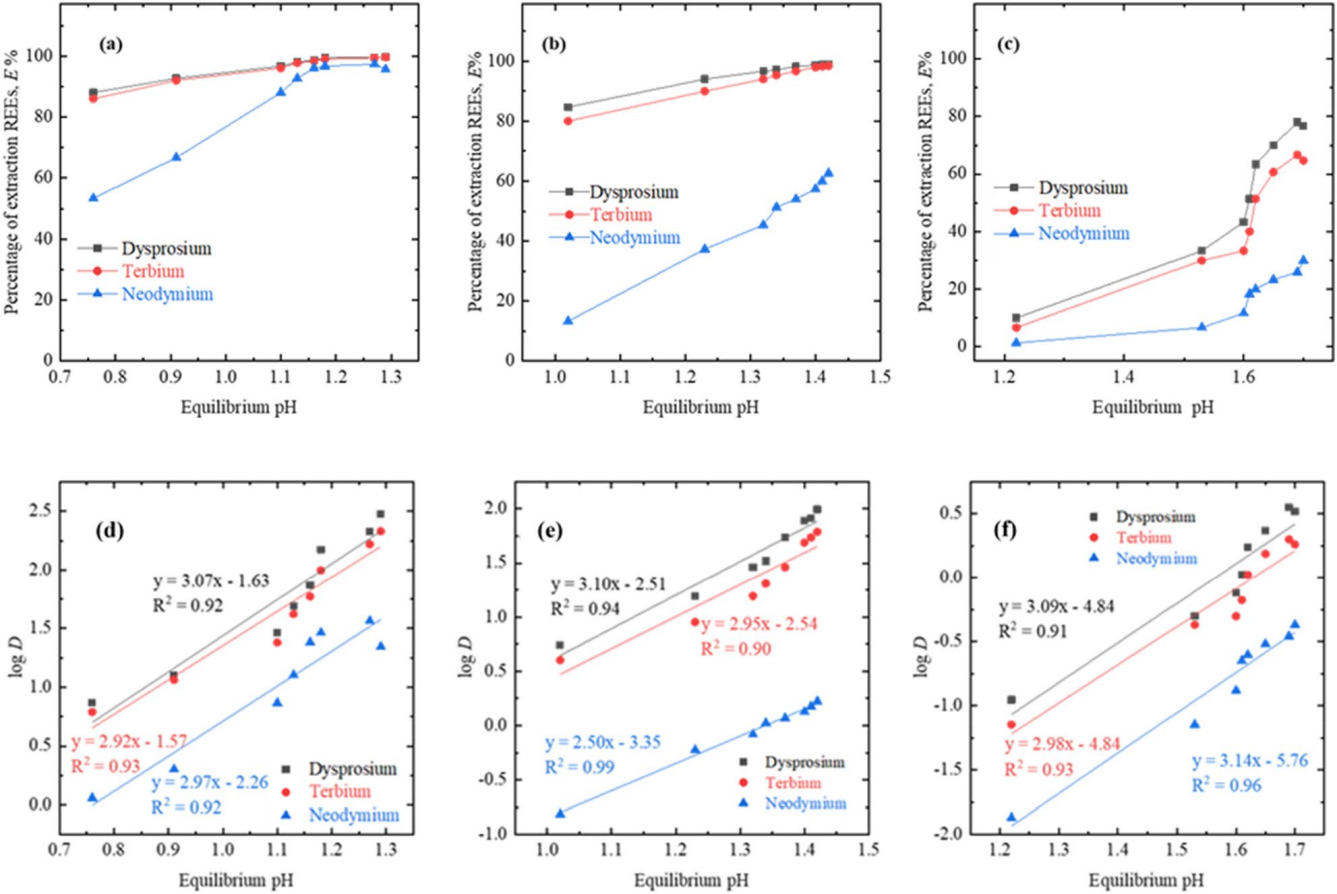
Effect of equilibrium pH using (**a**) D2EPHA, (**b**) PC 88A, and (**c**) Cyanex 272 as extractant systems for REEs extraction and Plots of log *D* as a function of equilibrium pH for (**d**) D2EPHA, (**e**) PC 88A, and (**f**) Cyanex 272. Experimental conditions: aqueous feed: 1500 mg/L Dy^3+^, 1500 mg/L Tb^3+^ and 1500 mg/L Nd^3+^, organic feed = 0.8 mol/L phosphorous based extractants, phase ratio A/O = 1:1, temperature = 298 K, and contact time = 60 min.

For all the three organo-phosphorus derivatives named PC 88A, D2EPHA, and Cyanex-272, the extraction of rare earth elements showed increasing trend when equilibrium pH of solution increased in the studied range of pH (0.76 to 1.7). The equilibrium pH range for D2EPHA was increased from 0.76 to 1.29, whereas it was increased from 1.02 to 1.42 with PC 88A and 1.22 to 1.70 for Cyanex 272, respectively. It was noticed that the extraction of REEs was negligible at initial pH 1.0 and the percentage of extraction augmented with the increase in initial pH (equilibrium pH) of aqueous phase (Fig. 1a–c). While comparing the extraction efficiency performances among the adopted reagents, extraction was the most effective with D2EPHA rather than with the other two extractants (PC 88A and Cyanex 272).

To ensure the cation exchange behaviour for the entire three employed acidic organic extractants while extracting REEs, slope analysis study was investigated and reported. Figure 1d–f indicated about the linearized plot of the equilibrium pH vs log *D* for all three metals (Dy, Nd, and Tb) resulting the slope value between 2.50 to 3.14 with either of three extractants. From the aforementioned data, it can be concluded that release of 3.0 mol of H^+^ from extractant phase to the aqueous phase for each mole of extraction of either of REE metals during liquid–liquid extraction process. The extraction mechanism of trivalent dysprosium, terbium and neodymium from the chloride solution with D2EPHA, PC 88A, and Cyanex 272 can be described in Eqs. 1–3 respectively.

### Effect extractant concentration

Various concentrations of extractants in the interval of 0.025 to 0.8 mol/L were prepared to examine the extractant concentration effect on the metal extraction efficiency of Dy, Tb and Nd from the chloride mediated solution. The liquid–liquid extraction experiment was conducted at phase ratio A:O = 1:1, at aqueous feed solution initial pH: 4 under ambient temperature 298 K (25 °C).

**Figure 2:**
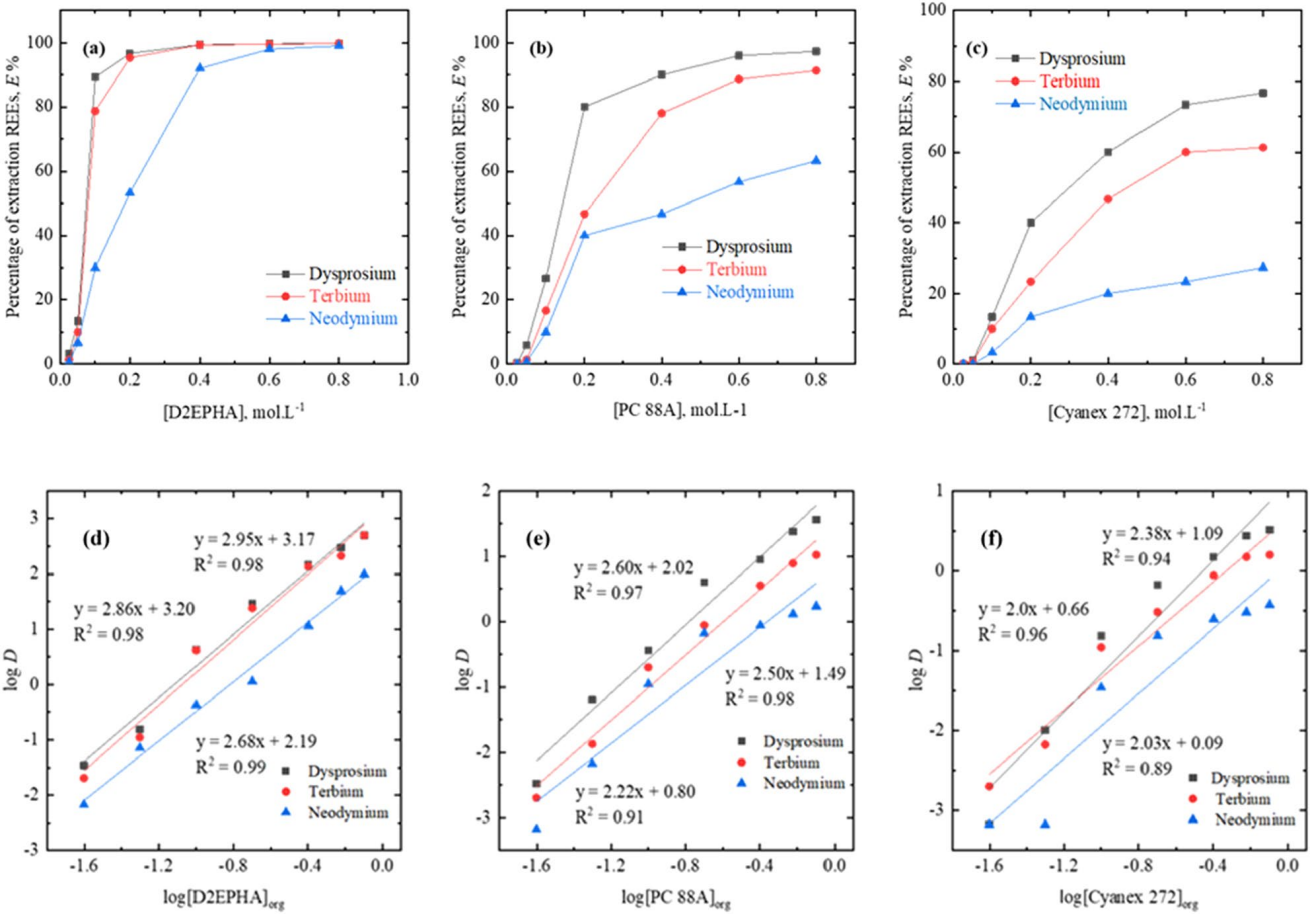
Extractant influence on rare earths extraction by using (**a**) D2EPHA, (**b**) PC 88A, and (**c**) Cyanex 272 and plots of log *D* vs log concentration of (d) D2EPHA, (**e**) PC 88A, (**f**) Cyanex-272. Experimental conditions: aqueous feed: 1500 mg/L Dy^3^^+^, 1500 mg/L Tb^3^^+^ and 1500 mg/L Nd^3^^+^, organic feed varied from 0.03 to 0.8 mol/L of phosphorous-based extractants, phase ratio A/O = 1:1, temperature = 298 K, and contact time = 60 min.

The performance of all three extractants on metal extraction of Dy, Tb and Nd was compared and results are as shown in Fig. 2a–c. The extractions of all three REEs using either of the organo-phosphrous reagents are promising. All the three REEs such as Dy, Tb and Nd were extracted 1 ± 0.5 to 99 ± 0.5% using 0.025 to 0.8 mol/L of D2EHPA. Whereas with PC 88A dysprosium (97%) and terbium (91%) were extracted more efficiently rather than neodymium (63% with 0.8 mol/L of PC 88A). On the other hand, with 0.8 mol/L of Cyanex 272 ~ 76% of dysprosium, 61% of terbium and 27% of neodymium were extracted. The extraction behavior of REEs with organo-phosphorus derivatives follows the series as D2EHPA > PC 88A > Cyanex 272.

The extraction behavior of REEs is consistent and in well agreement with the reported work^16^. The extractant D2EHPA appears to be very effective towards the extraction of REEs at mild acidic solution pH over other two reagents owing to its high loading ability. Based upon the extraction behavior while the adopting slope analysis method, all three REEs extraction mechanism either of the organo-phosphorous reagents is proposed.

To ascertain the association mechanism of REEs with organo-phosphorus reagents, the log *D* vs log D2EPHA (or) PC 88A (or) Cyanex 272 were plotted (Fig. 2d–f). Based on resulted slope values, the extraction mechanism was derived. As shown in the Fig. 2d the slope values of ~ 2.68 to 2.95 was obtained for extraction of the Dy, Tb and Nd, respectively using D2EHPA. In case of extraction of three rare earth elements: Dy, Tb and Nd with PC 88A, the slope values were of 2.60, 2.50 and 2.22, respectively (Fig. 2e). In case of Cyanex 272 the calculated slopes are 2.38 for Dy, 2.0 for Tb (or) Nd (Fig. 2f). Thus, it was evident that above the slope values are close to 2.0 in between 3.0 which reveal on the association of 2 to 3 mol of D2EHPA (or) PC 88A (or) Cyanex 272 per mole of either of the metal ion. The present study concluded that the cation exchange mechanism was strongly supported by the previous works^15–17^ while extracting these REEs from numerous aqueous media.

### Solvent extraction (liquid–liquid extraction) mechanism

The extraction of REEs (Dy, Tb and Nd) from the chloride medium is proposed according to the results of the effect of pH, the extractant variation on log *D* as described in the above sections. The plot of log *D* vs pH lead to have the resulted slope value indicating on the release of 2 to 3 number of H^+^ ions during complexation. In similar the association of 2 to 3 mol number of extractant was observed from the log *D* vs. log extractant plots (Fig. 2d–f). Accordingly, the general mechanism of extraction of REEs (Dy/Tb/Nd) with any organo-phosphorus reagents are proposed as given in the Eq. (1). The proposed mechanism seems well validated with the results obtained in this investigation.

1 $$n{ RE}_{ { }_{org}}^{3+}+n (H{A}_{2}{)}_{{ }_{org} \underset{.}{\iff }} n RE(H{A}_{2}{{)}_{n}}_{org}+n {H}_{ { }_{aq}}^{+}$$

2 $$K_{ex} = \frac{{RE (HA_{2} )_{n org } [H^{ + } ]_{ aq}^{n} }}{{n\left[ {RE^{3 + } ]_{aq} } \right[HA]_{ org}^{n} }}$$

3 $$K_{ex} = \frac{{D[H^{ + } ]_{ aq}^{n} }}{{[HA]_{ org}^{n} }}{ }$$

Taking logarithm of the Eq. (3) and rearranging, we get following the Eq. (4):

4 $$\mathrm{log}D=log{K}_{ex}+n log[HA{]}_{org}+n pH$$

where, REE = Dy (or) Tb (or) Nd, (HA_2_)_n org_ represents the dimeric form of organo-phosphorous extractants in Exssol D80 diluent system.

5 $$\% E = \frac{{(D \cdot (V_{org} /V_{aq} ))}}{{1 + (D \cdot (V_{org} /V_{aq} ))}}$$

where, %E = % Extraction, ‘*D*’ corresponds distribution coefficient = [RE]_org_/[RE]_aq_. V_org_ and V_aq_ are the organic and aqueous phases volume respectively.

The higher the value of ‘*D*’ at equilibrium, the higher is the extractability of the REE by the particular organo-phosphorus extractant. The percentages of extraction of REEs were calculated using Eq. (5).

The above proposed mechanism was derived based upon the slope analysis method as explained previously and it was again well supported by the mechanism proposed in the literature review^22–26^. Apart of which the association H^+^ ion and extractant species during complex formation of organo-phosphorous reagents are rational with this study and so as the works reported by researchers for extraction of numerous REEs using these reagents.

### Effect of diluents

The function of the diluents in solvent extraction is critical while dissolving the extractants by improving its extraction ability. It is well known from the literature, that the affinity of the metal in either phase (aqueous/organic) is governed by physical properties of the diluents including specific gravity, viscosity, dielectric constant and solubility parameters. For example, the viscosity of the extractant often retards the extraction efficiency and that lead to the inhibition of pure reagents (as such) in solvent extraction process. Thus, to have consistency of the metal extraction behavior, suitable diluents must be chosen. To investigate the diluent effect, five different diluents such as Exxsol D80, *n*-heptane, cyclo-hexane, xylene and toluene was adopted at two different concentrations: 0.1 mol/L and 0.8 mol/L for extraction of title rare earth elements using all the three P based extractants. The results of extraction efficiency for each of diluents are summarized in Table 1. Better extraction was obtained with aliphatic diluents than with aromatic diluents. The results showed that the chemical composition of the diluent might affect the extraction efficiency. The results presented emphasized a correlation between the dielectric constant and extraction efficiency. At low dielectric constants (1.92 to 1.98) for aliphatic diluents such as *n*-heptane and Exssol D 80 the extraction efficiency was maximized over the other diluents. There was apparent the increasing trend of REEs extraction as: Exxsol D80 > *n*-heptane > cyclo-hexane > xylene > toluene. Keeping in view of the higher extraction efficiency with Exxsol D80 as diluent, it was chosen as suitable one for subsequent extraction studies. Nevertheless, the above diluent is also an economical reagent with the prospective on its bulk level usage in continuous REEs solvent extraction processes.

**Table 1:** Effect of the diluents on the extraction of REEs with P based extractants.

Diluent	ε*	Extraction percentage			Extraction percentage			Extraction percentage		
		0.1 mol/L of D2EPHA			0.1 mol/L of PC 88A			0.1 mol/L of Cyanex 272		
		Dy	Tb	Nd	Dy	Tb	Nd	Dy	Tb	Nd
Xylene	2.28	78.16	58.85	11.74	50.25	27.51	3.04	20.71	13.18	9.29
N-heptane	1.92	96.31	90.67	12.25	77.57	57.06	11.74	34.60	21.56	10.56
Cyclo hexane	2.02	93.11	83.39	6.00	73.68	50.97	6.76	29.23	16.62	7.10
Toluene	2.38	40.96	18.41	1.00	74.60	53.58	8.62	13.48	4.94	0.85
Exxsol D80	1.98	97.98	94.48	48.85	76.59	55.5	41.18	38.14	19.27	17.31
		0.8 mol/L of D2EPHA			0.8 mol/L of PC 88A			0.8 mol/L of Cyanex 272		
Xylene	2.28	99.95	99.96	80.15	98.26	95.35	13.94	51.14	32.13	6.50
N-heptane	1.92	99.99	99.99	99.01	99.81	99.72	61.95	74.36	55.85	8.45
Cyclo hexane	2.02	99.98	99.98	98.13	99.70	99.41	40.72	71.51	51.86	4.81
Toluene	2.38	98.47	96.30	50.85	98.47	96.30	50.85	52.65	34.28	10.14
Exssol D80	1.98	99.97	99.97	99.71	99.89	99.86	75.05	76.75	59.44	40.00

*ε = Dielectric constant.

### Effect of temperature

**Figure 3:**
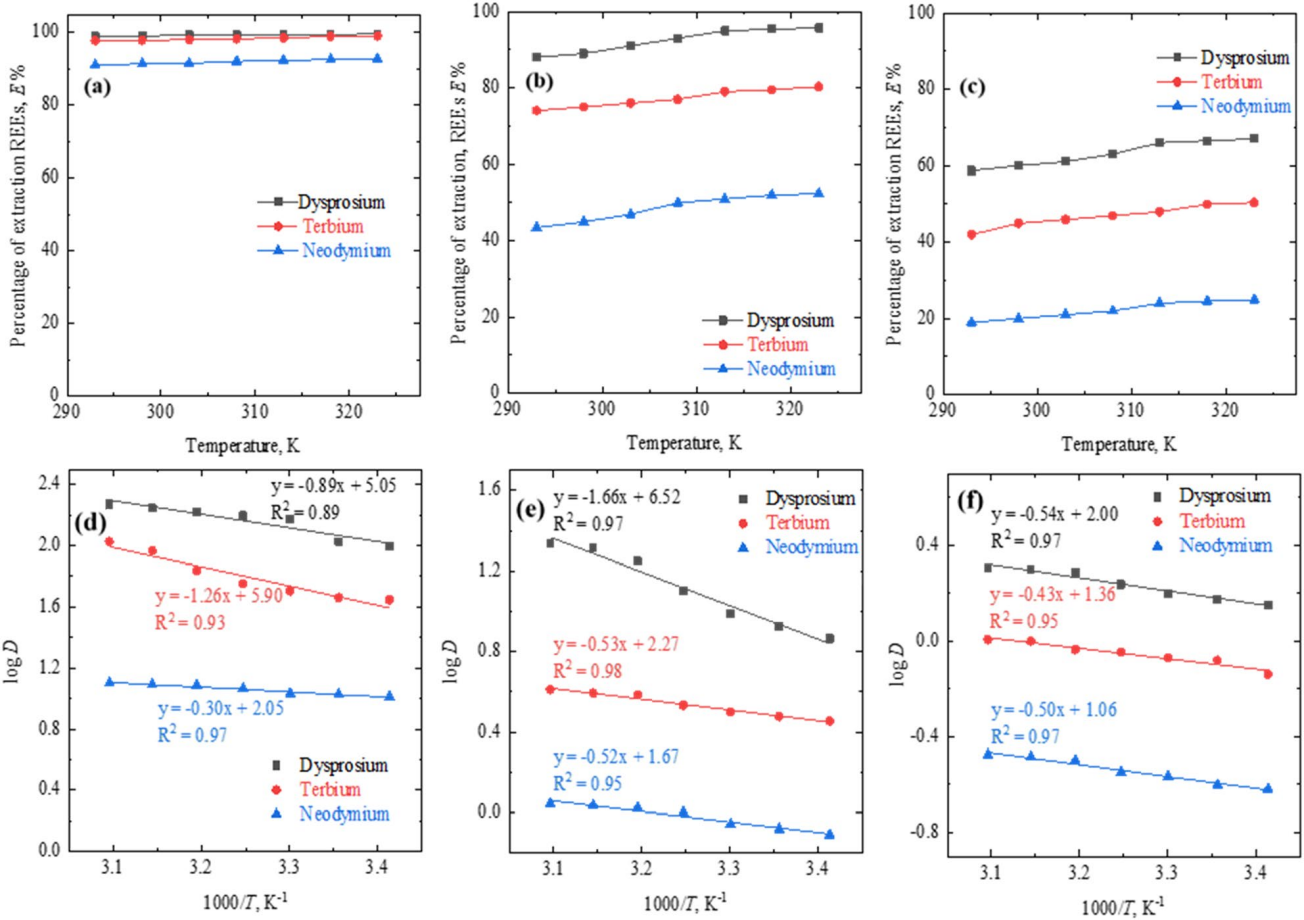
Effect of temperature on REEs extraction using (**a**) D2EPHA, (**b**) PC 88A, and (**c**) Cyanex 272 and plots of log *D* versus 1000/*T* (**d**) D2EPHA (**e**) PC 88A, (**f**) Cyanex 272. Experimental conditions: aqueous feed: 1500 mg/L Dy^3^^+^, 1500 mg/L Tb^3^^+^ and 1500 mg/L Nd^3^^+^, organic feed = 0.4 mol/L phosphorous based extractants, phase ratio A/O = 1: 1, initial pH 4.0, and contact time = 60 min.

The thermodynamic effect on the REEs Dy, Tb, Nd extraction using 0.4 mol/L concentration of either of the extractants (PC88A/D2EPHA/Cyanex 272) was studied in the temperature range of 293 K to 318 K at aqueous feed solution pH 4.0 and phase ratio of A:O = 1. The extraction efficiency of REEs (Dy, Nd and Tb) were increased while increasing the temperature of solution using all three P based extractants (Fig. 3a–c). The extraction behavior of the solvent reagents follows the sequence as D2EHPA > PC 88A > Cyanex 272 during the extraction of dysprosium, terbium and neodymium at studied temperature ranges. Amongst metals, Dy follows faster reaction rate followed by Tb and then Nd with either of the extractants.

To ascertain the extraction behavior the resulted data obtained at different temperature are fitted with thermodynamics Eq. (6) for evaluating the thermodynamic parameters such as ΔH and ΔS. Based on Eq. (6), the log *D* vs. log 1000/*T* was plotted (as shown in Fig. 3d–f) resulting the slope to determine the corresponding change in enthalpy. The + ΔH values of 17.04, 24.13, and 5.74 kJ/mol for Dy, Tb, and Nd while extracting with D2EHPA ensuring on the endothermic nature of extraction. The endothermic reaction of above metals have consistent numerical values and results are in well agreement with precious studies during extraction of REEs with acidic extractants while favoring the chemical reaction associated with the proposed solvent extraction process^26–29^. Furthermore, the change in free energy (ΔG) and entropy (ΔS) was evaluated based on the Eqs. (6), Eq. (7) and Eq. (8). As can be noticed from the results of Table 2, the values of ΔG (KJ/mol) = − 11.77 for Dy, − 9.54 for Tb and − 5.96 for Nd using D2EPHA showed the spontaneous nature of reaction during extraction of either of the metals. While, the standard Gibbs free energy values changed (ΔG = 0.42, 0.47, and 3.52 kJ/mol) indicates the Nd, Tb and Nd extraction reaction by PC 88A and Cyanex 272 respectively did not occur spontaneously. On the other hand, the positive ΔS (J/mol) values = 96.69 for Dy, 112.97 for Tb and 39.25 for Nd and reveled on the increasing in dis-order-ness of the reaction through the extraction process.

**Table 2:** Thermodynamics results for Dy, Tb and Nd extraction.

Thermodynamic variable*	D2EPHA			PC 88A			Cyanex 272		
	Dy	Tb	Nd	Dy	Tb	Nd	Dy	Tb	Nd
ΔH [KJ mol^−1^]*	17.04	24.13	5.74	31.78	10.15	9.95	10.34	8.23	9.57
ΔS [J K^−1^ mol^−1^]*	96.69	112.97	39.25	124.84	43.46	31.98	38.29	26.04	20.30
ΔG [KJ mol^−1^] 298 K*	− 11.77	− 9.54	− 5.96	− 5.42	− 2.80	0.42	− 1.07	0.47	3.52

*The variables for each element were calculated from Eqs. (6), (7), and (8).

6 $$\mathrm{\Delta H}=\frac{-2.303\mathrm{R\Delta logD}}{\Delta (1/\mathrm{T})}$$

7 $$\mathrm{\Delta G}=2.303{\mathrm{RTlogK}}_{\mathrm{ex}}$$

8 $$\mathrm{\Delta G}=\mathrm{\Delta H}-\mathrm{T\Delta S}\Rightarrow \mathrm{\Delta S}=\frac{\mathrm{\Delta H}-\mathrm{\Delta G}}{\mathrm{T}}$$

The extraction behaviour of these REEs (Dy, Tb and Nd) under studied temperature condition are promising and consistent with a significant increase in extraction efficiency. This extraction behaviour was strongly supported by the obtained thermodynamics results of this investigation as well as earlier works investigated related to the REEs extraction using various extractants^30–32^.

### FT-IR characterization analysis

**Figure 4:**
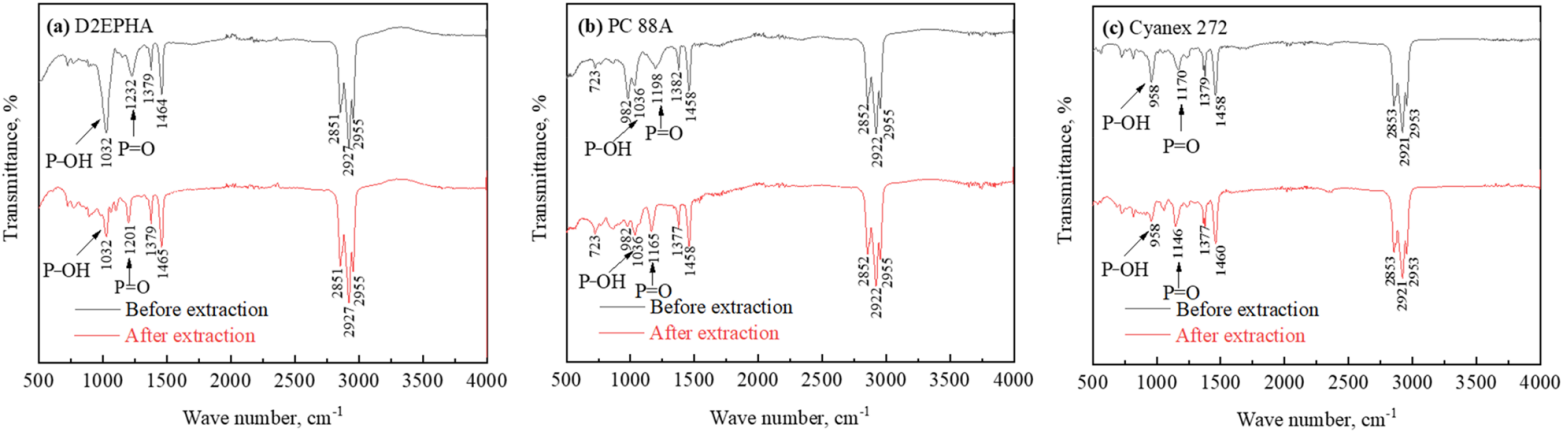
FT-IR spectra of the organic phases before and after extraction at maximum loading capacity of 0.8 mol/L of (**a**) D2EPHA / (**b**) PC 88A / (**c**) Cyanex 272.

To investigate the complex formation of all three rare earth elements Dy, Tb and Nd with organo-phosphorous reagents, the spectra of the organic phases was recorded before and after extraction by FT-IR and results are as shown in the Fig. 4.

The spectra showed P=O, and P–OH groups, which could indicate about the formation of REEs-extractant complexes. The absorption peaks at 1230–1170 cm^−1^ , 1036–958 cm^−1^ and 2851–2955 cm^−1^ were ascribed to the stretching vibration of P=O, P–OH and C–H bonds respectively in D2EPHA, PC 88A, and Cyanex 272 before extraction^33–35^.

Most of the absorption peaks maintained at their respective frequency level with extractants before and after extraction. However, P=O absorption peak positions are change during the complex formation. The P=O peak position shift from 1230 to 1201 cm^−1^ for loaded D2EPHA, while P−OH peak position remain constant at 1032 cm^−1^ but lower intensity, due to the complexation with REEs. The loaded PC 88A spectra showed a shift in that the absorption peak of P=O from 1198 to 1165 cm^−1^ supports on the participation of oxygen in P=O during the complex formation through coordination, but no variation the P−OH peak at 1036 cm^−1^. The spectra of loaded Cyanex 272 slight changed in the frequency of P=O peak from 1171 to 1146 cm^−1^ and P−OH band remained constant at 958 cm^−1^ with lower intensity compared with fresh reagent (before extraction).

The acidity of the P-based extractants follows order: D2EPHA > PC 88A > Cyanex 272, consequently the interaction between REEs and P based extractant is direct proportional to the acidity of P based extractants. D2EPHA proved the strongest tendency to react with REEs among the three P based extractant based on the efficiency. After solvent extraction, P-OH absorption peaks intensity is decrease in loaded organic of PC 88A, D2EPHA and Cyanex 272.

### McCabe–Thiele diagrams of REEs extraction

To investigate the required stage numbers for attaining quantitative extraction of rare earth elements dysprosium, terbium and neodymium from the mixed aqueous feed solution, the A/O phase ratio was diversified in the range of 1:5 to 5:1. The REEs concentration obtained for the aqueous solution and organic phases for each of the organo-phosphorous reagents plotted in either of the axis. The McCabe–Thiele diagram was constructed at 0.05 mol/L D2EHPA for extraction of Tb and Dy where the co-extraction was noticed to be of ~ 26.7% of Nd. In this way the flow sheet was designed to separate out entire Tb and Dy while retaining the major concentration of Nd at the raffinate phase.

As shown in Supplementary Data file Fig. S2a and b, 3 and 4 stages are theoretically needed for a complete extraction of Dy and Tb, respectively at A:O = 1 for the entire extraction of either of the REEs metals from the mixed solution. To confirm the above prediction, 4 stages counter current simulation study (CCS) was performed and the above results were validated (Supplementary Data file Fig. S2c). After 4 cycles CCS study the substantial raffinate solution reads to contain < 1 mg/L of Dy, < 1 mg/L of Tb and 1100 mg/L of Nd, ensuring on > 99.9% extraction of Dy and Tb with ~ 26.7% of Nd co-extraction into the loaded organic phase.

Keeping in view of obtaining clean separation with enriched extraction of Nd from the resulted raffinate phase, further the extraction isotherm plot was assembled at 0.8 mol/L of D2EHPA and results are as shown in Supplementary Data file Fig. S3a. This plot predicted on quantitative extraction of Nd at A:O = 4:1in two stages. To validate the above isotherm predication for extraction of Nd, CCS study was examined and that resulted more than 99.9% Nd extraction at the proposed condition (Supplementary data file Fig. S3b). The above loaded organic was subjected to stripping study for further enrichment with regeneration of the D2EHPA for further use at the proposed flow diagram.

### Stripping studies

Likewise to the extraction study, stripping is equally significant for regeneration of adopted extractant in the process and so as the enrichment of extracted metal into the stripped solution for achieving its clean separation. There are several in organic-acids, alkalis and salts are employed for stripping of REEs from the loaded-organic phase^36–40^, but these regents have less stripping ability even at higher concentration level. To overcome such issue and to attain quick regeneration of extractant organic green reagents like oxalic acid is preferred as suitable reagent. The other advantage of the adoption of oxalic acid is of its strong stripping ability vis-à-vis formation of corresponding precipitated oxalate product after aging of solution. In this way the number of processing- step as well as time could be minimized.

To investigate the concentration of oxalic acid on the stripping of neodymium from LO-D2EHPA, the concentration varied from 0.03 to 1.0 mol/L. Prior to stripping study an adequate quantity of loaded organic has been generated. The stripping study was examined for 30 min under the shaker-incubator and after equilibration, the two phases for disengagement were allowed. The stripped solutions were collected and since the partial precipitation was observed just after stripping for each stripped solutions. Therefore to ensure the stripping efficiency of oxalic acid at the studied concentration ranges, the regenerated organic phase was stripped further with 1.0 mol/L HCl (4 times) followed by subsequent dilution for analyzing the neodymium content retained at the LO phase after stripping. The results of stripping efficiency with oxalic acid at varying concentration level is shown in Table 3 from this investigation, oxalic acid 0.5 mol/L effectively stripped the Nd content (> 99.9%) from the loaded organic phases and thereafter a plateau was achieved.

**Table 3:** Stripping studies of neodymium using oxalic acid (0.8 mol/L of D2EPHA loaded Nd = 4400 mg/L).

Experiment number	Concentration of oxalic acid, mol/L	Neodymium extraction, %
1	0.03	41.2
2	0.06	56.3
3	0.10	97.1
4	0.25	98.3
5	0.50	99.9
6	0.75	98.7
7	1.00	98.4

However, in order to ascertain the higher Nd enrichment during stripping, the loaded organic was used for stripping isotherm study as described in the following section. The Nd-loaded D2EHPA (0.8 mol/L) was subjected to the stripping isotherm study using 0.5 mol/L of oxalic acid by varying SO:SS (SO = spent organic and SS = strip solution) ratio from 1:5 to 5:1 and the entire volume of the solution was kept stable during the stripping process. The key objective of this investigation was to improve the enrichment of Nd content in to the stripped solution for substantial recovery of Nd product. The stripping McCabe–Thiele plot illustrated that is necessary 2 numbers of stage at phase ratio of SO:SS = 4:1 for the complete stripping of Nd from 0.8 mol/L of D2EHPA loaded organic phase (Supplementary Data file Fig. S4a). This predicts on fourfold enrichment of Nd in stripping stage. To validate above proposition, 2 cycle counter current simulation (CCS) was studied at the mentioned isotherm condition. The resulted SO was read to contain < 0.01 mg/L of Nd ascertaining on more than 99.9% of Nd stripping from the loaded organic phase leading to generate an enriched concentrate bearing ~ 17,600 mg/L of Nd (Supplementary Data file Fig. S4b).

### Neodymium compound preparation

**Figure 5:**
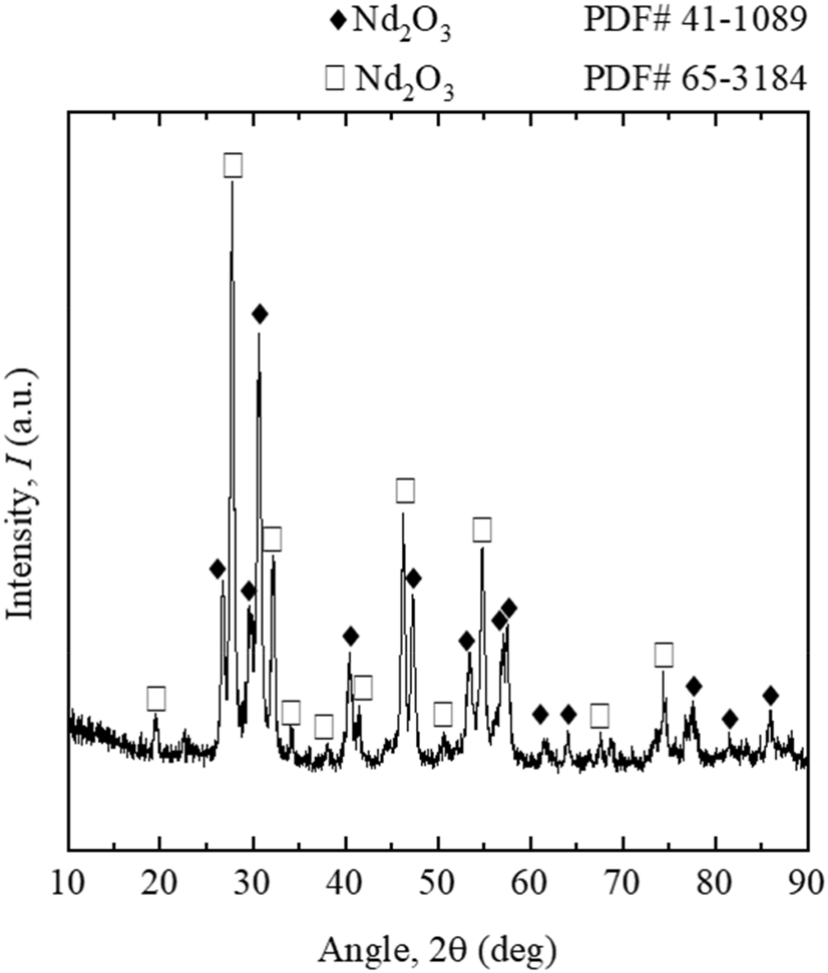
XRD of precipitated product after-calcination from the stripped solution.

The enriched stripped liquor appeared in precipitated form, but to ensure the complete precipitation solution it was kept on aging (3 h). The precipitate was dried out to free water content at 383 K to obtain neodymium oxalate, the color of it was light purple powder (Supplementary Data file Fig. S5a). To obtain pure form of neodymium oxide from the neodymium oxalate was calcinated for 1 h under a muffle furnace at the treated temperature of 1173 K (Supplementary Data file Fig. S5b). The resulted high pure neodymium oxide was confirmed from the XRD results as shown in Fig. 5.

**Figure 6:**
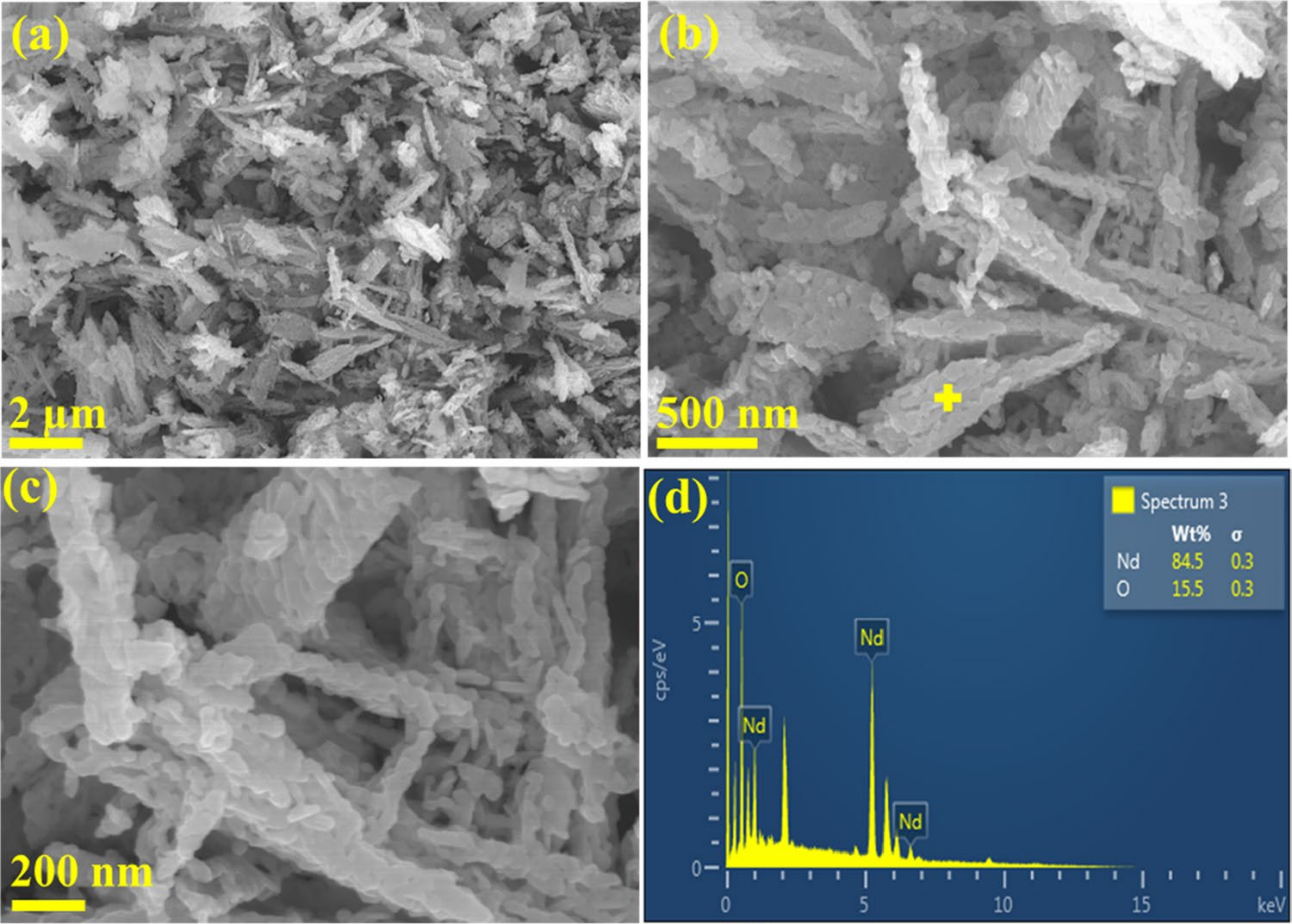
FE-SEM images (**a**–**c**) of neodymium oxide and its EDS spectra from corresponding point (**d**).

SEM–EDS images of the samples showed that the particles are fused together and formed as long sheets. The size of the fused particles are about 30 to 50 nm. The sheets have porous structure. SEM–EDS results confirmed that the particles have the neodymium (Nd) and oxygen (O) elements (unassigned peaks in EDS spectra are corresponding to the coating material-Pt). Moreover, Nd and O are in 84.5 and 15.5 wt% respectively (Fig. 6).

SEM–EDS results confirmed that the particles have the Nd and O elements (unassigned peaks in EDS spectra are corresponding to the coating material-Pt). Moreover, Nd and O are in 84.5 and 15.5 wt% respectively.

**Figure 7:**
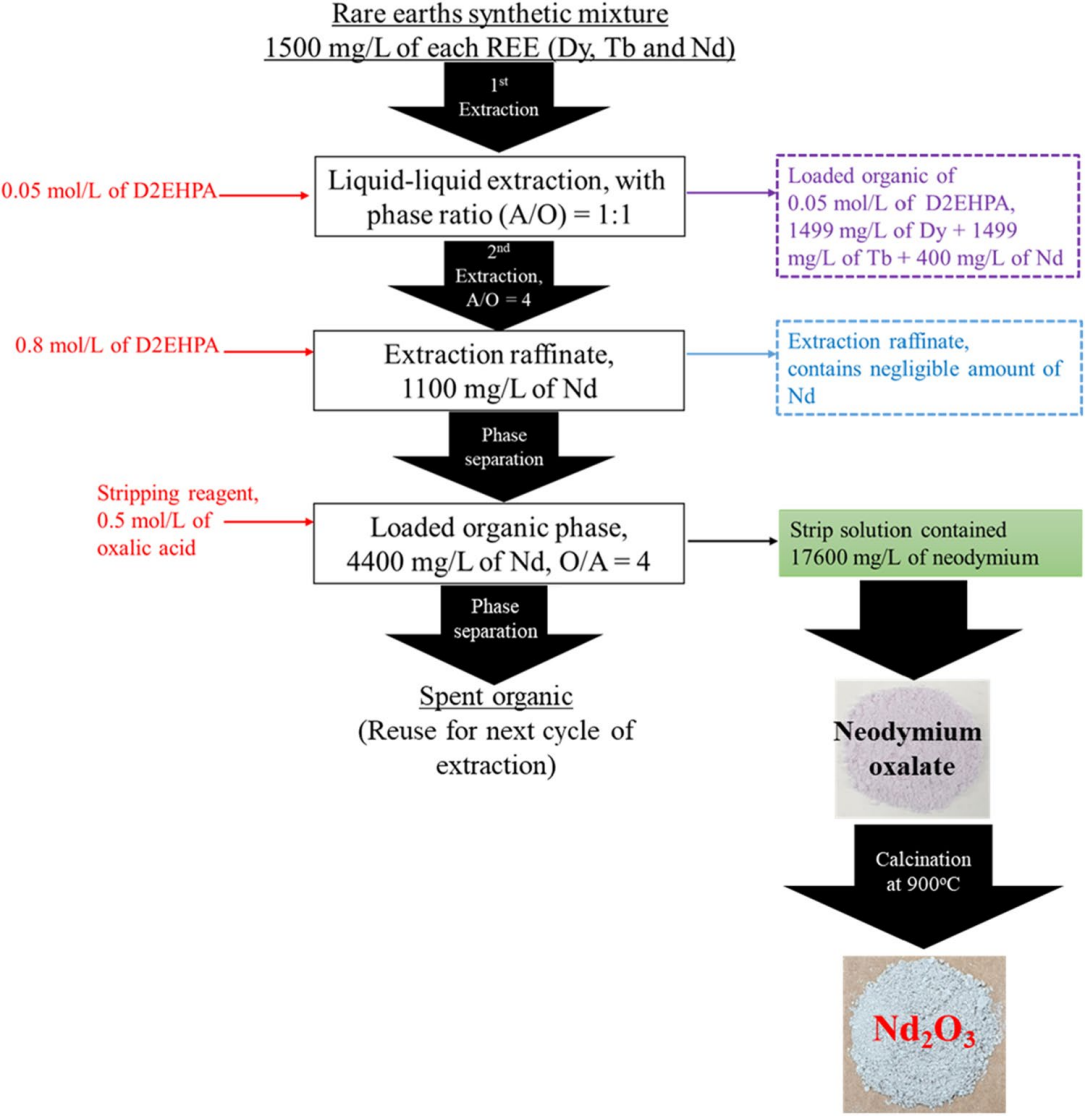
Overall environmentally friendly hydrometallurgical process for neodymium extraction and possible separation from dysprosium and terbium.

The present developed method for REEs extraction and possible separation was compared with former methods on REEs processing by solvent extraction and better separation possibilities with enriched metal loading towards neodymium metals was achieved (Table 4). The developed hydrometallurgical processing of neodymium extraction and possible separation from other associated elements such as dysprosium and terbium was presented and summarized in Fig. 7.

**Table 4:** Summary and comparison of the reported literature on rare earths processing.

Rare earths	Description of the process	Chemical reaction/note	Reference
Nd, Dy, Y	Separation of Nd, Dy and Y by solvent extraction from chloride solution using D2EHPA and EHEHPA in n-heptane	$RE{E}^{3+}+m{H}_{2}{A}_{2}=REE{A}_{3}{(HA)}_{2m-3}+3{H}^{+}$ $RE{E}^{3+}+3{Cl}^{-}+ {m}^{\mathrm{^{\prime}}}{H}_{2}{A}_{2}=REEC{l}_{3}{(HA)}_{{2m}^{\mathrm{^{\prime}}}}$ $REEC{l}_{s}^{{\left(3-s\right)}^{+}}+m{H}_{2}{A}_{2}=$ $REEC{l}_{s}{A}_{3-s}{(HA)}_{2m-3+s}+\left(3-s\right){H}^{+}$	^3^
Nd, Dy	Recovery Process Development for the Rare Earths from Permanent Magnet Scraps Leach Liquors using D2EPHA in kerosene	${M}_{(aq)}^{n+}+n{(HA)}_{2(org)} \stackrel{.}{\leftrightarrow } M{A}_{n}{(HA)}_{n (org)}+n{H}_{(aq)}^{+}$	^4^
Nd, Pr	Separation and recovery of Nd and Pr from permanent magnet scrap using D2EPHA	${M}_{aq}^{+3}+3 ({HR)}_{2 (org)}\underset{.}{\leftrightarrow } M{R}_{3}{3HR}_{(org)}+3{H}^{+}$	^5^
Nd	Solvent extraction of Nd from a chloride solution using PC 88A, Cyanex 272, and Cyanex 302 in kerosene	$N{d}^{3+}+3{(HA)}_{2}+2{A}^{-} \stackrel{.}{\leftrightarrow } Nd{A}_{3}.5HA+ {H}^{+}$	^6^
Nd, Dy	Selective recovery of REEs from Md-base permanent magnets dissolved HNO_3_ solution using TODGA and Cyanex 923	$TODGA+FeC{l}_{3}+HCl \underset{.}{\leftrightarrow } TODGA.{H}^{+}. {FeCl}_{4}^{-}$ $3TODGA. {H}^{+}.{FeCl}_{4}^{-}+ {Nd}^{3+}+ {3Cl}^{-} \underset{.}{\leftrightarrow }$ $[(TODGA{)}_{3}{Nd]}^{3+} (FeC{l}_{4}^{-}{)}_{3}+3HCl$	^7^
Y, Sm, Eu, Tb, Dy	Extraction behavior of REEs from acidic chloride media using tetra-butyl di-glycol-amide in kerosene 1-octanol	${Ln}_{\left(aq\right)}^{3+}+n{TBDGA}_{(0)}+ {3Cl}_{(aq)}^{-} \stackrel{.}{\leftrightarrow } Ln{(TBDGA)}_{N} {Cl}_{{3}_{(0)}}$ REE extraction can be efficient without the use of high acid concentration	^8^
Ce, La, Nd, Pr	Solvent extraction of the lighter lanthanide metal ions using D2EPHA, PC 88A, Cyanex 272 and 301 in kerosene	${M}^{3+}+ {m(HR)}_{2} \stackrel{.}{\leftrightarrow } {MR}_{3}{(HR)}_{2M-3}+3{H}^{+}$ Pr > Nd > Ce > La pH 1–5 with all organophosphorus extractants Cyanex 301 and 272 = low and medium extraction	^9^
Pr, Nd	Separation of Pr and Nd from chloride solution using Cyanex 272 and mixture of extractants in escaid 110 in chloride medium	Organic phase: mixture of 1 mol/L 10 pct saponified Cyanex 272 and 0.5 mol/L TBP aqueous phase: La—781.5 mg/L, Pr—119.1 mg/L and Nd—333.9 mg/L, organic phase: 0.001–2 mol/L Cyanex 272, A/O ratio = 1	^10^
Nd, Pr, Dy	Recovery of REEs from neodymium magnet waste using glycolic, maleic, and ascorbic acids with D2EPHA in solvent 70	$L{n}^{3+}+x (HR{)}_{2} \underset{.}{\leftrightarrow }Ln{R}_{3}{(HR)}_{2X-3}+3{H}^{+}$ $L{n}^{3+}+3 RCO{O}^{-}+3TBP \underset{.}{\leftrightarrow } Ln {(TBP)}_{3}+ {(RCOO}^{-})$	^11^
La, Nd, Sm, Gd	Synergistic extraction of rare earths by mixture of HDEHP and HEH/EHP diluted in kerosene in sulfuric acid medium	$R{E}_{(a)}^{3+}+2{H}_{2}{B}_{2}{.}_{(0)}{H}_{2}{L}_{2}{.}_{(0)} \stackrel{{K}_{12}}{\leftrightarrow } RE (H{B}_{2}{)}_{2} (H{L}_{2}{)}_{(0)}+3{H}^{+}$ ${M}^{3+}+m{(HR)}_{2} \stackrel{.}{\leftrightarrow } {MR}_{2}{(HR)}_{2M-3}+ {3H}^{+}$	^12^
Nd, Dy, Pr, Gd, Co, and B	Separation of heavy REEs from light REEs from a neodymium magnet leachate using D2EPHA in solvent 70 in sulfuric acid medium	The separation between the HREEs and the LREEs, the best separation factors with 0.3 M D2EHPA in hexane. Under such conditions, almost all the Dy and Gd were extracted from	^13^
La, Nd and Ce	Solvent Extraction of Light Rare Earth Ions Using D2EHPAin kerosene from Nitric Acid and Sulphuric Acid Solutions	The utmost extraction percentage of La, Nd and Ce (99.4pct , 99.7pct and 100 pct respectively) was extracted from 0.1 M HNO3 using 1.0 M D2EHPA ${\mathrm{Ln}}^{3+}+\mathrm{x}{(\mathrm{HR})}_{2}\stackrel{.}{\leftrightarrow }\mathrm{ Ln}{\mathrm{R}}_{3}{(\mathrm{HR})}_{2\mathrm{x}-3}+3{\mathrm{H}}^{+}$ ${\mathrm{Ln}}^{3+}+3{\mathrm{RCOO}}^{-}+3\mathrm{TBP }\underset{.}{\leftrightarrow }\mathrm{ Ln}{(\mathrm{TBP})}_{3}+ {(\mathrm{RCOO}}^{-})$	^14^
Dy and Nd	Recovery of Dy and Nd from permanent magnet scraps leach liquor dissolved in sulfuric acid using PC 88 A in kerosene	${\mathrm{M}}_{(\mathrm{aq})}^{\mathrm{n}+}+ {\mathrm{n}(\mathrm{HA})}_{{2}_{(\mathrm{org})}}= {\mathrm{MA}}_{\mathrm{n}}{(\mathrm{HA})}_{\mathrm{n}}+{\mathrm{nH}}_{(\mathrm{aq})}^{+}$ Time = 5 min, pH 3, A/O = 1.5:1 SF = 492	^15^
Dy, Tb and Nd	D2EHPA showed high extraction ability over PC 88A followed by Cyanex 272 The role of temperature appears to be critical while enhancing the extraction rate and the resulted positive ΔH values 11.74, 5.85, and 9.48 kJ/mol Overall 17.6 g/L of Nd was recovered from 1.5 g/L of aqueous feed solution	$n{ RE}_{ { }_{org}}^{3+}+n (H{A}_{2}{)}_{{ }_{org} \underset{.}{\iff }} n RE(H{A}_{2}{{)}_{n}}_{org}+n {H}_{ { }_{aq}}^{+}$	Present method

## Experimental

### Materials and reagents

The synthetic solution containing the desired concentration of REEs dysprosium (Dy), terbium (Tb) and neodymium (Nd) was prepared by dissolving the REEs oxides with hydrochloric acid and distilled water. All these stock rare earth oxides were procured from High Purity Chemicals Company from Japan. The acid and alkali reagents such as HCl and NaOH used for pH adjustment of aqueous solutions are of analytical grade (Merck, Pvt Ltd). The three organo-phosphorous (P) based commercial extractants such as di(2-ethyl-hexyl) phosphoric acid (D2EHPA), 2-ethyl-hexyl phosphonic acid mono-2-ethyl-hexyl ester (PC88A) and bis(2,4,4-tri-methyl pentyl) phosphinic acid (Cyanex 272) were procured and taken to uses as it is without making further purifications. The pure form of D2EHPA was supplied by Aldrich and PC 88A was obtained by Daihachi Chemical Industry, Japan whereas Cyanex 272 was received from Cytec. The requested concentration of either of these extractants were prepared with suitable dilution using Exxsol D80 as diluent manufactured by ExxonMobil Chemical.

### Experimental and analytical equipment(s)

The solvent extraction experiments were realized in a controlled temperature shaking incubator (Jeio Tech, Model SI-300/300R/600/600R). The entire experimented aqueous samples bearing REEs metals were analyzed by Inductive Coupled Plasma (iCAP 6000 Series, Thermo Scientific, USA). The solution pH was measured in a pH meter (Orion star, Model A215). The organic samples (D2EHPA/PC 88A/Cyanex 272) and its REE-loaded at maximum loading capacity were characterized by Fourier Transform Infrared Spectroscopy (Model Nicolet 6700 FT-IR spectrometer Thermo Scientific Corp.). FT-IR samples were prepared by the procedure of spreading one drop on germanium disk (optically transparent in the range 6000–450 cm^−1^). The subsequent final product neodymium oxide was characterized to ascertain its phase by X-ray diffraction analysis in a X-Ray diffractometer (XRD: RIGAKU, RINT 2000, Cu-Kα radiation) diffractometer with Cu-Kα radiation (k = 1.5418 Å). The morphology, size and composition was examined by field-emission scanning electron microscopic (FE-SEM, S-4800 Hitachi), and energy dispersive X-ray spectroscopic (EDS, FE–SEM) measurements.

### Solvent extraction (liquid–liquid extraction) procedure

The REEs bearing solution was equilibrated with organophosphorus reagents under a shaker incubator with a shaking speed of 250 rpm, while keeping either of the solution [aqueous (A) and organic (O)] equal with phase contact time of 60 min (except where otherwise provided) using a separating funnel. In case of isotherm studies A/O ratios were varied within 1:5 to 5:1 and in which entire volume of phases were kept stable. In each experiment study during extraction, dilute HCl or NaOH was added to control the pH to the desired value. Subsequently, the equilibration both the phases were subjected to phase disengagement followed by separation to read the resulted equilibrium pH. The raffinates of each samples were diluted to the needed times using dilute HCl and followed by their analysis of REE metals. To ensure mass balance, the loaded organics were sieved by 1PS (phase separating filter paper) and the stripped with 2 mol/L HCl (4 times) prior to its analysis. The REEs concentration in the organic phase was obtained by the subtraction of the concentrations in the raffinate before and after extraction. The experiments were performed at room temperature except for the thermodynamic investigation. The experimental error was between the agreed distribution ratios ± 3%.

## Conclusions

The following conclusions were drawn from the developed methodology for REEs such as dysprosium, terbium and neodymium from chloride synthetic leach solutions by hydrometallurgical techniques.

1. Amongst the solvent regents adopted in this study, D2EHPA showed high extraction ability over PC 88A followed by Cyanex 272.
2. The proposed extraction mechanism derived through the slope analysis method confirmed on releasing of 2 to 3 mol of H^+^ ion with association of 2 to 3 mol of extractant in most cases studies.
3. FT-IR characterization results of organic phase samples examined before and after extraction study and it was confirmed that, complex formation with rare earths.
4. The role of temperature performs to be vital while magnifying the extraction rate and the resulted positive ΔH values while favouring about the endothermic nature of reaction during extraction process.
5. The McCabe–Thiele plot constructed using 0.05 mol/L D2EHPA showed preferential extraction of Dy and Tb than that of Nd, ensuring on selective Nd separation prospective from the mixed chloride mediated solution.
6. A flow sheet for extraction and enrichment of Nd by D2EHPA was developed with A:O = 4:1 at extraction stage followed by stripping with oxalic acid at LO:SS = 4:1 yielded the final stripped solution leads to bear ~ 17,600 mg/L of neodymium oxalate.
7. Overall Nd enrichment was enhanced up to 11.7 folds with subsequent recovery of neodymium oxalate followed by calcination of the final pure product as Nd_2_O_3_.The single phase of either of the product was confirmed from XRD analysis results. SEM–EDS results confirmed that the particles have the Nd and O elements; those are in 84.5 and 15.5 wt% respectively.

## Supplementary information

Supplementary Information.
