# Supplementary material for: Environmentally friendly comprehensive hydrometallurgical method development for neodymium recovery from mixed rare earth aqueous solutions using organo-phosphorus derivatives
Source: Sci Rep. 2020 Oct 9;10:16911. doi: 10.1038/s41598-020-74041-9 (PMC7547677; doi:10.1038/s41598-020-74041-9)
Supplement: Supplementary file 1 — Supplementary Information. [file 41598_2020_74041_MOESM1_ESM.docx]

Supplementary data

Environmentally friendly comprehensive hydrometallurgical method development for neodymium recovery from mixed rare earth aqueous solutions using organo-phosphorus derivatives

Verónica Cristina Arellano Ruiz^a,b^, Rambabu Kuchi^a^, Pankaj Kumar Parhi^c^*, Jin-Young Lee^a,b*^, Rajesh Kumar Jyothi^a,b^*

^a^Convergence Research Center for Development of Mineral Resources (DMR), Korea Institute of Geoscience & Mineral Resources (KIGAM), Daejeon 34132, Korea

^b^Department of Resources Recycling, University of Science and Technology (UST), Daejeon 34113, Korea

^c^ Department of Chemistry, Fakir Mohan University, [Balasore](https://en.wikipedia.org/wiki/Balasore) 756 089, [Odisha](https://en.wikipedia.org/wiki/Odisha), [India](https://en.wikipedia.org/wiki/India)

Correspondence and requests for materials should be addressed to R.K.J. (email: [rkumarphd@kigam.re.kr](mailto:rkumarphd@kigam.re.kr)) or J.-Y.L. (email: jinlee@kigam.re.kr) or P.K.P (email: parhipankaj@gmail.com)


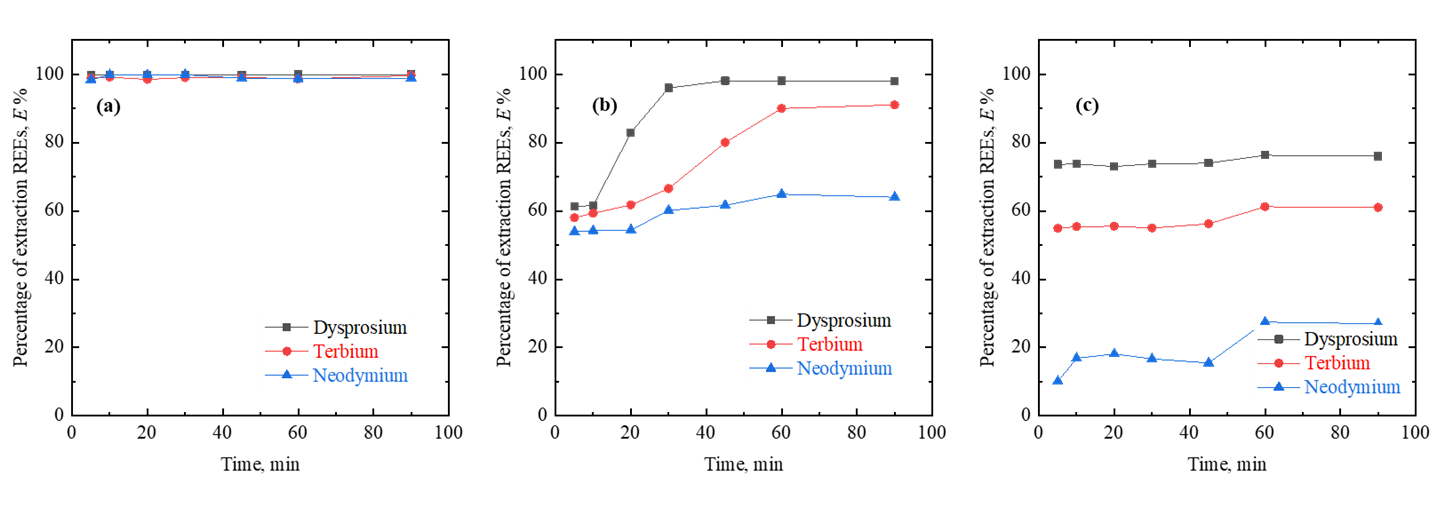


Fig. S1. Efficacy of metal extraction as a function of contact time of (a) D2EPHA, (b) PC 88A, and (c) Cyanex 272 for dysprosium, terbium, and neodymium extraction. Experimental conditions: aqueous feed: 1500 mg/L Dy^3+^, 1500 mg/L Tb^3+^ and 1500 mg/L Nd^3+^, organic feed = 0.8 mol/L phosphorous based extractants, phase ratio A/O = 1: 1, initial pH = 4.0, temperature = 298 K, contact time varied from 5 to 90 min.


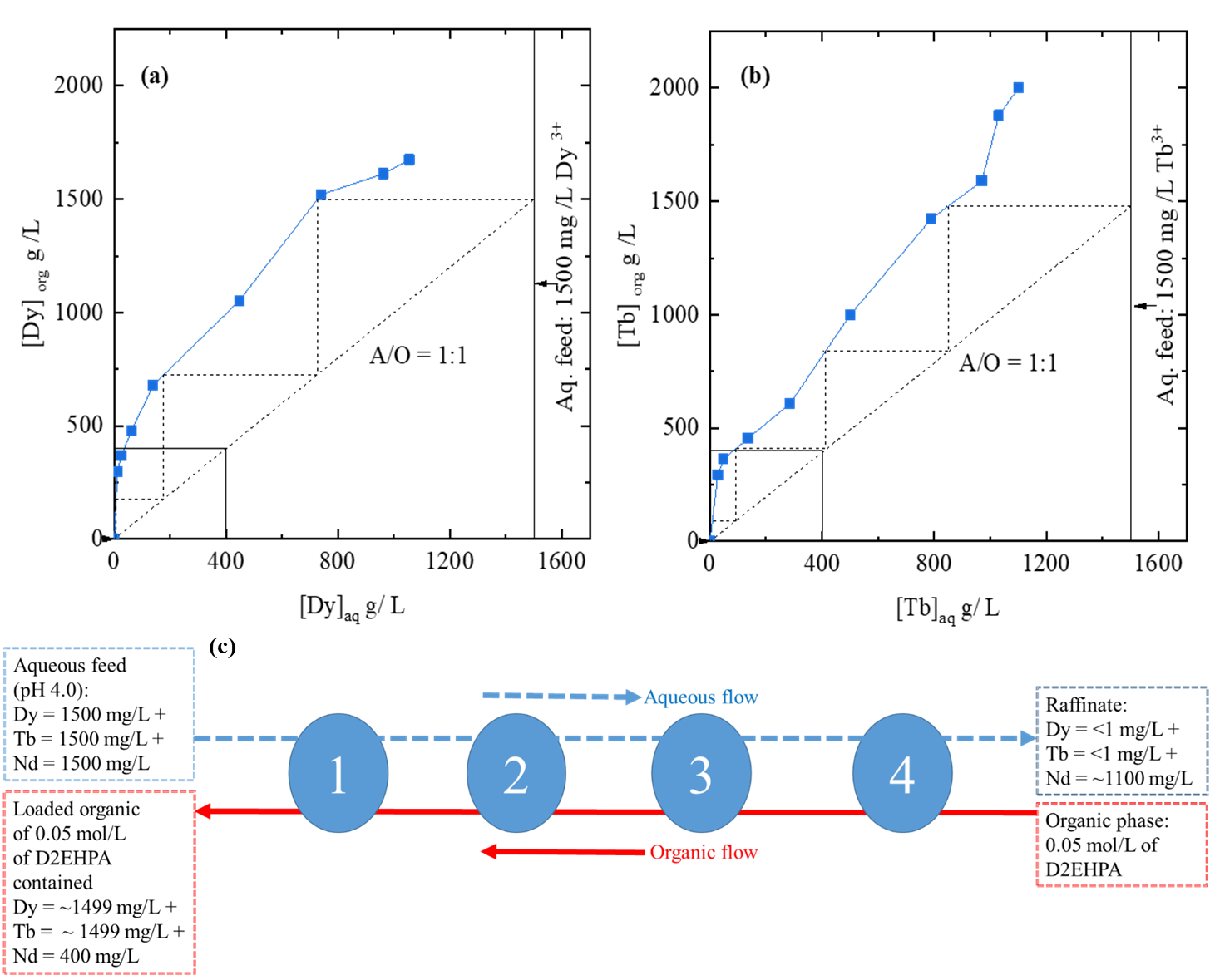


Fig. S2. McCabe-Thiele plot for extraction of (a) Dy and (b) Tb using 0.05 mol/L of D2EPHA, (c) Counter-current simulation for extraction study of the REEs using 0.05 mol/L of D2EHPA at initial pH 4 (aqueous feed solution) Aq. = aqueous feed, Experimental conditions: aqueous feed: 1500 mg/L of Dy^3+^, 1500 mg/L of Tb^3+^ and 1500 mg/L of Nd^3+^, temperature = 298 K, time = 60 min, initial pH of aqueous feed = 4.


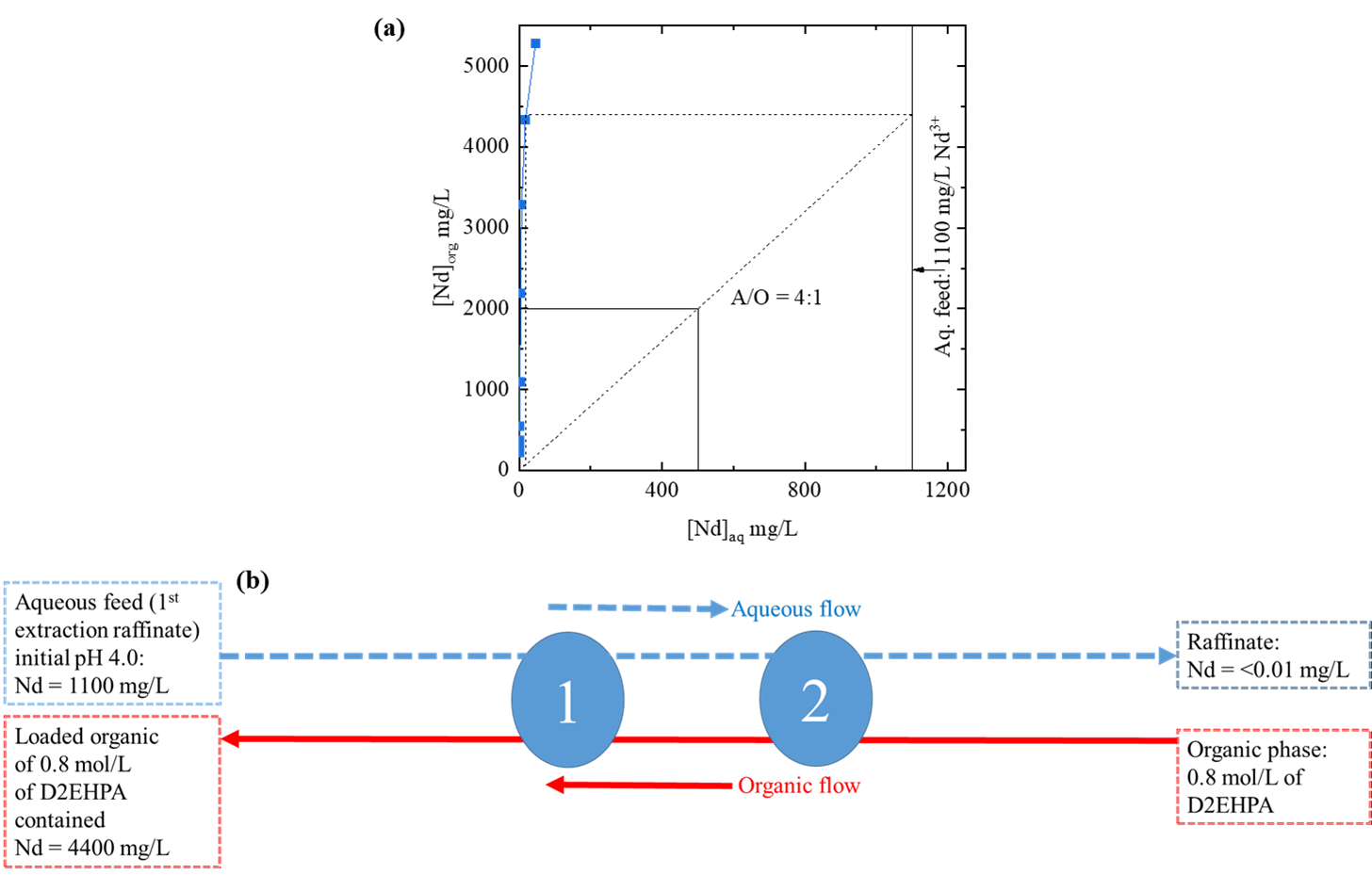


Fig. S3. (a) McCabe-Thiele plot for extraction of neodymium with 0.8 mol/L of D2EPHA. (Aq. = aqueous feed) Experimental conditions: aqueous feed = 1100 mg/L Nd^3+^, temperature = 298 K, time = 60 min, initial pH of aqueous feed = 4. (b) Counter-current simulation for extraction study of the neodymium from 1^st^ extraction raffinate solutions by using 0.8 mol/L of D2EHPA at initial pH 4 (aqueous solution). Experimental conditions: phase ratio (A/O) = 4:1, time = 60 min, temperature = 298 K.


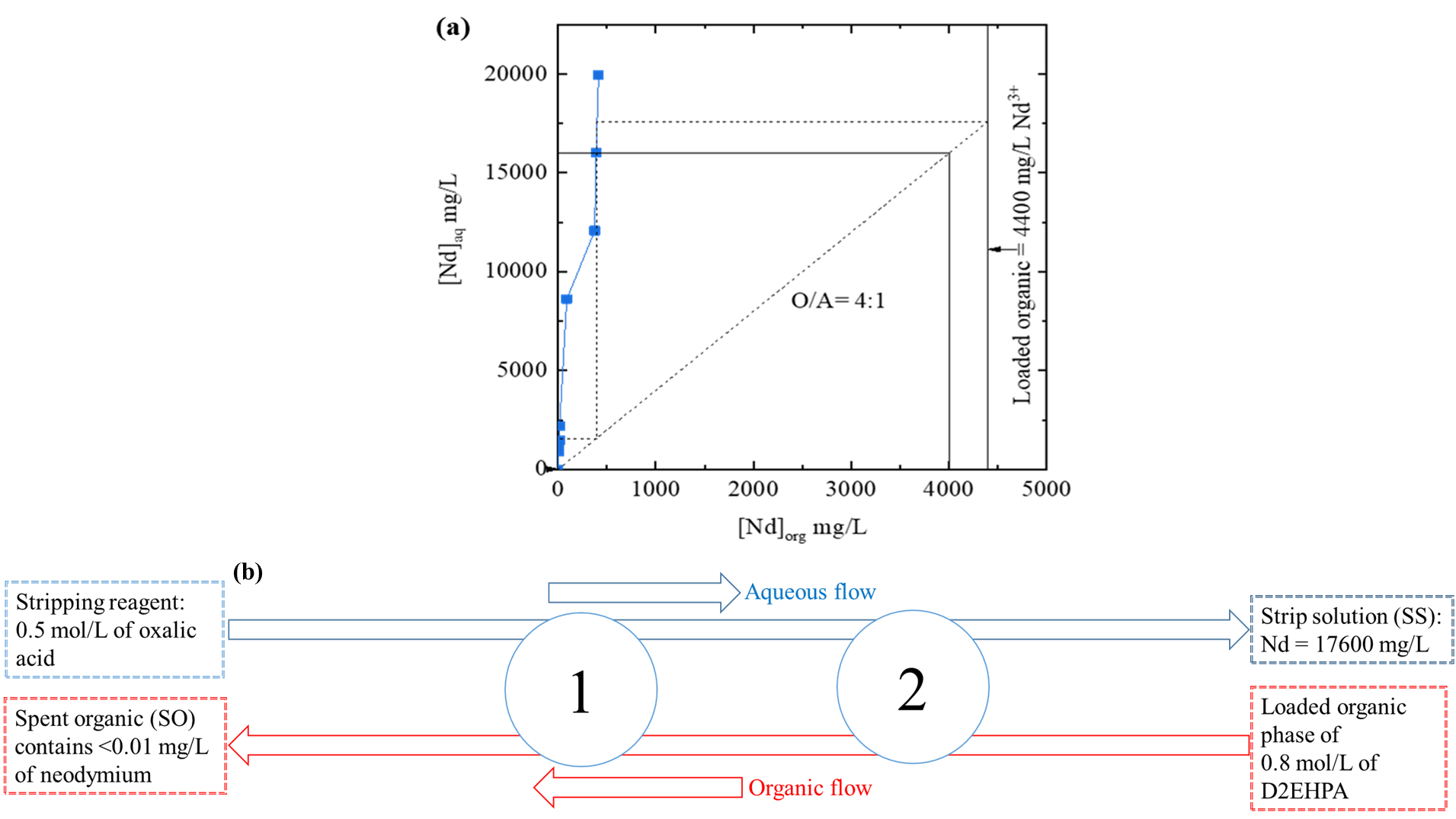


Fig. S4. (a) McCabe-Thiele plot for stripping (back extraction of the targeted metal from loaded organic phase) of neodymium from 0.8 mol/L of D2EPHA loaded organic (LO) phase. (b) Counter-current simulation for stripping (back extraction of the targeted metal from loaded organic phase) study of the neodymium with 0.5 mol/L oxalic acid. Experimental conditions: temperature = 298 K, time = 30 min, striping reagent (aqueous phase) = 0.5 mol/L oxalic acid.


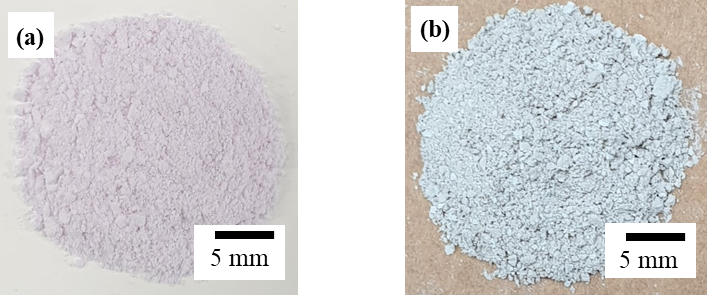


Fig. S5 Photographs of **(a)** neodymium oxalate **(b)** neodymium oxide
